# Sexual Outcomes after Conservative Management for Patients with Localized Penile Cancer

**DOI:** 10.3390/curroncol30120765

**Published:** 2023-12-17

**Authors:** Simone Cilio, Antonio Tufano, Gabriele Pezone, Pierluigi Alvino, Gianluca Spena, Savio Domenico Pandolfo, Paola Del Prete, Claudio Amato, Rocco Damiano, Andrea Salonia, Riccardo Autorino, Alessandro Izzo, Francesco Passaro, Sisto Perdonà

**Affiliations:** 1 Urology Unit, Department of Neurosciences, Reproductive Sciences and Odontostomatology, University of Naples “Federico II”, 80131 Naples, Italy; 2 Department of Maternal-Infant and Urological Sciences, Policlinico Umberto I Hospital, “Sapienza” Rome University, 00161 Rome, Italy; 3 Scientific Directorate, Istituto Nazionale Tumori di Napoli, IRCCS “G. Pascale”, Via M. Semmola, 80131 Naples, Italy; 4 Dipartimento di Chimica e Tecnologia del Farmaco, Sapienza University of Rome, Piazzale Aldo Moro 5, 00185 Rome, Italy; 5 Urology Unit, Magna Graecia University of Catanzaro, 88100 Catanzaro, Italy; 6 Unit of Urology/Division of Experimental Oncology, URI, IRCCS Ospedale San Raffaele, 20141 Milan, Italy; 7 Department of Urology, Rush University Medical Center, Chicago, IL 60637, USA; 8 Uro-Gynecological Department, Istituto Nazionale Tumori di Napoli, IRCCS “G. Pascale”, Via M. Semmola, 80131 Naples, Italy

**Keywords:** penile cancer, penile neoplasm, localized penile cancer, penile-sparing surgery, glansectomy, wide local resection, quality of life, sexual dysfunction, erectile function, IIEF-5, CSFQ

## Abstract

Background: Men with localized invasive penile cancer (PC) can be treated with organ-sparing treatments with different functional and aesthetical outcomes. Thus, the aim of this study is to investigate sexual outcomes in patients with PC confined to the glans that underwent wide local excision (WLE) vs. glansectomy with urethral glanduloplasty. Methods: Complete data from 60 patients with PC were analyzed at our institution from 2017 to 2022. Patients were asked for personal habits and clinical features. PC was assessed with a clinical visit and imaging techniques. At the outpatient follow-up visit or phone call, all patients compiled the Changes in Sexual Function Questionnaire (CSFQ) and the International Index of Erectile Function in its short 5-item form (IIEF-5). Erectile function (EF) impairment was categorized using Cappelleri’s criteria. Results: Overall, 34 patients with PC confined to the glans (c ≤ T2N0) were included. Of those, 12 underwent WLE and 22 underwent glansectomy with urethral glanduloplasty. Using multivariable logistic regression, glansectomy (OR: 3.49) and diabetes (OR: 2.33) were associated with erectile disfunction (IEEF < 22). Meanwhile, using multivariable linear regression analysis, younger patients (Coeff: −2.41) and those that underwent glansectomy (Coeff: −7.5) had a higher risk of sexual function impairment, according to the CSFQ. Conclusions: Patients with PC ≤ T2N0 that underwent WLE have better outcomes in terms of sexual functioning than the patients who underwent glansectomy and uretheral gladuloplasty. Further research is needed to clarify the outcomes of penile-sparing surgery, to inform patients in pre-surgical counseling more comprehensively, and to meet their post-operative expectations more effectively.

## 1. Introduction

Penile cancer (PC) is a rare tumor with an incidence ranging from 0.94/100,000 men in Europe to 2% of men in Southern America, Asia, and Africa [1]. More than half of cases recognize human papillomavirus infection as the main etiological factor, followed by phimosis, tobacco exposure, poor hygiene, and multiple urinary tract infection [2,3,4]. According to clinical presentation, surgical treatment for localized PC can be divided into demolitive (partial or radical penectomy) or conservative treatments, such atopical therapy, laser ablation, wide local excision (WLE), and glansectomy [1,5]. Conservative treatments are usually reserved for patients with localized tumors (c ≤ T2) with the aim of eradicating the entire cancer, obtaining free margins of excision and sparing the organ [6]. In this scenario, urologists are increasingly favoring penile-sparing approaches due to their remarkable functional outcomes and significant preservation of patients’ physical and psychosexual wellbeing [7]. Interestingly, Maddineni et al. evaluated the effects of PC and its treatment on psychosexual, social, and QoL experiences, concluding that PC had a negative impact on wellbeing in up to 40% of patients; almost 50% of men that underwent radical surgeries show symptoms of a psychiatric illness and up to two thirds experience an impairment of sexual function [8].

However, despite the evidence that an appropriate pre-operative education and post-operative management can improve sexual function in men that underwent surgery due to PC [9,10], the current literature only reports few studies regarding the comparison of penile-sparing approaches for localized PC in terms of patients’ post-operative expectations [11]. Consequently, our study aims at analyzing, from the perspective of a single tertiary oncological referral center, the sexual function in men that underwent conservative surgery (WLE vs. glansectomy with urethral glanduloplasty) for localized PC confined to the glans.

## 2. Materials and Methods

We retrospectively analyzed the data from 60 patients that attended our institution between January 2017 and September 2022 for newly diagnosed PC. A detailed medical history was collected for all subjects and data on health-significant comorbidities were scored using the Charlson Comorbidity Index (CCI). At the pre-operatory medical assessment, patients were asked about their recreational habits, such as smoking habits (categorized as follows: non-smokers, ex-smokers, and active smokers) and current or personal history of phimosis. Further clinical data that were collected comprised hypertension, defined by the measurement of arterial systolic pressure ≥ 140 mm/Hg and/or arterial diastolic pressure ≥ 90 mm/Hg, or by the daily use of one or more antihypertensive drugs; diabetes, defined as glycated hemoglobin (Hb1Ac) ≥ 6.5% or daily use of one or more antidiabetic drugs; cardiovascular disease, defined as follows: acute myocardial infarction and/or surgical treatment of coronary artery disease, angina, cerebrovascular accidents (i.e., stroke, transient ischemic attack), congestive heart failure, aortic aneurysm; and nonfatal cardiac arrhythmias, defined as a minimum of an arrhythmia requiring cardiovascular treatment.

For the specific aim of the study, we only selected patients with PC confined to the glans and with a negative lymph node status (cTa-T2N0) according to clinical and imaging findings. We therefore compared the clinical characteristics of patients who underwent WLE vs. glansectomy with urethral glanduloplasty. Specifically, patients with pTa and T1 lesions ≤ 2 cm underwent WLE, while patients with pT1 > 2 cm lesions, pT2 lesions, or multiple glans lesions underwent glansectomy with urethral glanduloplasty.

After a median of 12 months from the surgery, at the outpatient follow-up visit or via a phone call, all patients were invited to compile the Changes in Sexual Function Questionnaire (CSFQ) and the 5-item version of the International Index of Erectile Function (IIEF-5) [12,13]. Specifically, the CSFQ is a 14-item questionnaire assessing 5 domains of sexual function, such as overall satisfaction, sexual desire in terms of both overall interest and frequency in desiring sexual intercourse, sexual arousal, and orgasm-related satisfaction. Lastly, IIEF-5 was used to assess the erectile function after penile-sparing surgery; EF impairment was categorized by using Cappelleri’s criteria.

Data collection followed the principles outlined in the Declaration of Helsinki; all patients signed informed consent, agreeing to deliver their own anonymous information for future studies. This study was approved by the “Istituto Nazionale Tumori—IRCCS Fondazione Pascale” Ethical Committee.

### 2.1. Surgical Procedures

Patients with localized tumors with a maximum diameter inferior to 2 cm, underwent WLE. The adopted procedure consisted of the removal of neoplastic tissue together with a safe macroscopical margin from two to five millimeters. The margin-free status was confirmed through laboratory frozen section analysis.

In the case of patients with a glans-localized tumor with higher dimensions, or multiple localization, glansectomy with urethral glanduloplasty was the chosen treatment option. In this procedure, the glans is totally resected, the distal margins of corpora cavernosa are exteriorized and sutured ventrally under the urethra, thus restoring an appearance of the balanopreputial sulcus. Subsequently, the urethral stump is spatulated in its ventral portion, the exposed mucosa is then sutured to the albuginea covering the distal corpora cavernosa. The coloration of the urethral mucosa confers a glans-like appearance and ensures a certain degree of local sensibility.

### 2.2. Statistical Analysis

Statistical analysis consisted of three steps. First, means (SD) and medians (IQR) or frequencies and proportions were reported for continuous or categorical variables, respectively. To compare the statistical significance of the differences in the distribution of continuous or categorical variables among the two groups (WLE vs. glansectomy), the Mann–Whitney U test and Chi-squared or Fisher’s exact tests were performed, respectively. Second, multivariable logistic regression models identified the potential predictors of erectile dysfunction (IIEF-5 < 22) in men with localized PC that underwent WLE vs. glansectomy. Third, multivariable linear regression models were used to assess the changes in sexual function according to CSFQ questionnaire.

The RStudio graphical interface v.0.98 for R software environment v.3.0.2 (http://www.r-project.org, accessed on 13 November 2023) was used to perform statistical analyses. All statistical tests were two-sided with a significance level set at *p* < 0.05.

## 3. Results

A total of 34 patients were included in this study with a mean (SD) age of 65.2 (9.5) years. Of those, 79.4% were married and 32.4% were current smokers. The median (IQR) tumor size was 16 (11–19) mm and no positive surgical margins were observed. Moreover, only two Clavien 3 complications (i.e., meatal stenosis) were recorded during the follow-up visits.

Overall, 12 patients underwent WLE and 22 were treated with glansectomy and urethral glanduloplasty. Table 1 details the descriptive characteristics of the patients according to the penile-sparing procedure utilized (WLE vs. glansectomy, respectively). For pre-operative variables, no statistical differences were depicted between the two groups, except for tumor size (11 vs. 18 mm; *p* = 0.005). On the questionnaires that were administered 12 months post-surgery, patients that underwent glansectomy showed worse IIEF-5 and CSFQ scores (20.1 vs. 17.0, *p* = 0.02, and 48.6 vs. 40.3, *p* < 0.001, respectively).

With a median follow-up of 32.5 months, two recurrences, both in the glansectomy group, were observed, with a median time to local recurrence of 18.5 months. All the recurrences were treated with repeat organ-sparing procedures.

Multivariable logistic regression analysis showed that patients that underwent glansectomy (OR: 3.49) and had diabetes (OR: 2.33) were more likely to be associated with erectile disfunction (IEEF < 22) (Table 2). On the other hand, the multivariable linear regression analysis performed on younger patients (Coeff: −2.41) that underwent glansectomy (Coeff: −7.5) presented a higher risk of sexual function impairment, according to the CSFQ (Table 3).

## 4. Discussion

This cross-sectional study analyzed real-life data from 34 patients treated at a single referral oncological center for localized PC of the glans. Of all, 12 patients underwent WLE and 22 glansectomy. Between the two cohorts, no statistical baseline characteristics differences were recorded according to their clinical history and check-up, with the exception of the length of their hospital stay. Regression models used in our analysis showed that patients with diabetes and that underwent glansectomy with urethral glanduloplasty were more likely to develop ED, with higher impairment of sexual function in younger patients. Moreover, according to post-operative complications, we did not record any major complications, and WLE depicted better results than glansectomy, considering that the two cases of meatal stenosis were reported after the latter procedure.

Since Pizzocaro et al. released the first version of the European Association of Urology (EAU) guidelines on PC management in 2009, medical researchers showed higher interest in elucidating the emasculating consequences of operative approaches in terms of psychological, physical, and sexual morbidity [14]. Indeed, non-radical techniques showed very low rates of cancer recurrence after the collection of clean surgical margins of <10 mm from the cancer without the loss of the organ [15]. In this setting, a literature review by Kamel et al. in 2017 showed that local recurrence rates are higher in patients that underwent an organ-sparing surgery than penile amputation. However, the authors declared that most of these recurrences can be managed by performing other non-radical surgeries, allowing patients to enjoy better sexual function and a better overall QoL [16]. With the same aim, Li et al. analyzed data from a cohort of 32 patients that underwent penile-sparing surgeries for localized low-grade PC. After a median follow-up period of 27 months, they reported that organ-sparing techniques were appropriate options for eradicating cancer and preserving both sexual and urinary function without significantly increasing the risk of cancer recurrence [17].

In 2018, Wan et al. analyzed aesthetical, sexual, and urinary functions and the QoL of a series of fifteen patients, eight that underwent WLE and seven that underwent partial penectomy. They found that both organ-sparing techniques could achieve good outcomes in terms of aesthetical, sexual, and urinary functions and the QoL. Particularly, patients that underwent WLE depicted better, even if not statistically significant, outcomes in terms of overall satisfaction. However, in patients treated with organ-sparing techniques, the authors recorded an important loss of orgasmic function, which appeared otherwise less severe in patients that underwent WLE, with respect to men that underwent partial penectomy [18]. Moreover, as shown in the current literature, the decline of the overall sexual satisfaction in men treated with demolitive approaches is related to the reduction of penile dimensions [19]. However, patients that undergo organ-sparing surgeries without losses in penile length may report a decline in sexual desire. Indeed, Scarberry et al. found, in a retrospective analysis of patients treated with penile-preserving approaches, that half of them reported no sexual activity or even denied the existence of their own sexual desire [20]. In this scenario, according to the CSFQ our data showed that, among patients that underwent penile-sparing surgeries for PC, the ones treated with glansectomy with urethral glanduloplasty have a higher risk of declined overall sexual function in comparison to patients that underwent WLE.

Lastly, with the aim of finding the best option to treat localized PC with the minimum loss of tissues, Sedigh et al. (2015) compared the post-operative outcomes of 41 patients diagnosed with localized PC that underwent WLE vs. glansectomy or partial amputation according to the cancer patients’ sexual expectations. The authors concluded that, among the most conservative treatments, WLE leads to better sexual outcomes, with preserved erectile function and less post-operative complications compared to glansectomy [21]. Thus, our analysis is consistent with the previous findings, affirming that WLE could represent the best conservative approach for the treatment of localized primary PC to preserve sexual and erectile functions, without causing higher cancer recurrence rates. Interestingly, previous studies assessed how younger men diagnosed with PC showed worst outcomes in terms of nodal involvement and overall cancer-specific survival [22,23]. In this regard, our analysis showed that a younger age is associated with worse outcomes only in the setting of erectile function. Indeed, we recorded the absence of statistically significant differences in terms of age in men with a tumor stage ≤ 1 who underwent WLE vs. T stage ≤ 2 who underwent glansectomy with urethral glanduloplsty.

As a first strength point, our study fits in an open and debated scenario regarding the best surgical management for locally non-advanced PC. Moreover, our findings could help in ideating and developing further surgical trials with the aim of elucidating clinical and functional outcomes in the management of PC. Lastly, the homogeneity of the baseline characteristics of this study’s population is one of the major strengths of our study, as it eliminates the possible baseline intra- and intercohort biases.

On the other hand, our study is not exempt from limitations. Firstly, the retrospective nature of this study is a limitation. Consequently, we encourage further research on this topic to explore post-surgical outcomes in patients that underwent penile-sparing surgery for localized PC during the pre-operatory assessment in more depth. Furthermore, due to the rarity of the disease, the cohort of the included patients comprised a small sample size, which could devalue the statistical power of our findings. In this regard, prospective, multicenter, randomized trials could be designed to compare these two different conservative treatments. Lastly, we did not have access to the data on baseline sexual functions nor the data regarding patients’ emotional status (such as depression, anxiety, etc.), which may have a great impact on patients diagnosed with PC. Thus, the absence of reliable baseline information do not allow us to analyze intracohort outcomes before and after surgery. However, none of the patients declared pre-operative ED or other sexual function disorders, nor previously diagnosed alterations of emotional status.

## 5. Conclusions

Penile-sparing procedures can be considered as a suitable management choice for selected patients, taking into account the established disease risk factors. In our analysis, the patients with localized PC that underwent WLE had better outcomes in terms of sexual function when compared to patients that underwent glansectomy with urethral reconstruction, providing excellent local oncological control. Nevertheless, individuals eligible for these procedures should undergo vigilant monitoring and adhere to the follow-up requirements. Further research is needed to explore psychological and oncological long-term outcomes in this patient population in order to enhance the selection of patients during pre-surgical counseling and to meet their post-operative expectations more effectively.

## Figures and Tables

**Table 1:** Characteristics of the study population.

	Overall *n* = 34	Wide Local Excision *n* = 12	Glansectomy *n* = 22	*p* Value
Age, mean (SD)	65.2 (9.5)	63.2 (10.8)	66.4 (8.7)	0.35
Smoking, *n* (%)				
Never	9 (26.5)	3 (25.0)	6 (27.3)	
Current	11 (32.4)	4 (33.3)	7 (31.8)	0.98
Former	14 (41.2)	5 (41.7)	9 (40.9)	
Marital status, *n* (%)				
Married	27 (79.4)	9 (75.0)	18 (81.8)	0.63
Single/divorced	7 (20.6)	3 (25.0)	4 (18.2)	
Diabetes, *n* (%)	4 (11.8)	1 (8.3)	3 (13.6)	0.65
Hypertension, *n* (%)	18 (52.9)	5 (41.7)	13 (59.1)	0.33
CVD, *n* (%)	3 (8.8)	2 (16.6)	1 (4.5)	0.23
ASA score, median (IQR)	2 (2–2)	2 (2–2)	2 (2–2)	0.51
CCI, median (IQR)	3 (2–4)	3 (2–4)	3 (2–4)	0.65
Complications, n (%)				
Clavien 1–2	0 (0)	0 (0)	0 (0)	0.27
Clavien ≥ 3	2 (5.9)	0 (0)	2 (9.1)	
Positive margins, n (%)	0 (0)	0 (0)	0 (0)	-
Tumor size, mm, median (IQR)	16 (11–19)	11 (10–18)	17 (15–21)	**0.005**
12 months post-operative IIEF-5 score, mean (SD)	18.6 (3.4)	20.1 (2.3)	17.0 (3.6)	**0.02**
12 months post-operative CSFQ score, mean (SD)				
Pleasure	3.0 (1.1)	3.4 (1.2)	3.0 (1.1)	0.35
Sexual desire/frequency	8.1 (1.5)	8.3 (1.6)	7.9 (1.3)	0.23
Sexual desire/interest	11.5 (2.4)	11.8 (2.2)	11.3 (2.4)	0.45
Arousal/excitement	9.8 (2.2)	11.0 (1.9)	9.0 (2.1)	**0.004**
Orgasm/completion	9.5 (3.1)	11.3 (3.4)	8.0 (3.1)	**<0.001**
Total	42.2 (3.5)	48.6 (3.6)	40.3 (2.5)	**<0.001**

CVD: cardiovascular diseases; ASA: American Society of Anesthesiologists; CCI: Charlson Comorbidity Index. Bold = statistically significant.

**Table 2:** Multivariable logistic regression for predicting erectile disfunction according to the IIEF questionnaire (defined as IIEF < 22).

	O.R.	95% C.I.	*p* Value
Age	1.74	(0.87–2.33)	0.11
ASA score	1.23	(0.76–1.87)	0.22
WLE	-	-	
vs.			
Glansectomy	3.49	(2.87–6.78)	**<0.001**
Hypertension	1.45	(0.97–1.98)	0.07
Diabetes	2.33	(1.15–2.97)	**<0.001**

ASA: American Society of Anesthesiologists; WLE: wide local excision. Bold = statistically significant.

**Table 3:** Multivariable linear regression in predicting Changes in Sexual Function Questionnaire (CSFQ).

	β Coefficient	95% C.I.	*p* Value
Age	−2.41	(−4.71; −1.12)	**0.02**
WLE	-	-	
vs.			
Glansectomy	−7.52	(−13.11; −4.21)	**<0.001**
Hypertension	−3.45	(−14.04; 5.03)	0.52
Diabetes	−10.12	(−18.14; 1.28)	0.07

WLE: wide local excision. Bold = statistically significant.

## Data Availability

The data presented in this study are available upon request from the corresponding author. The data are not publicly available due to privacy restrictions.
